# Influence of sex and functional status on the value of serum steroid profiling in discriminating adrenocortical carcinoma from adrenocortical adenoma

**DOI:** 10.3389/fendo.2024.1435102

**Published:** 2024-09-18

**Authors:** Yan Weng, Ju-Ying Tang, Xiao-Yun Zhang, Diao-Zhu Lin, Ying Guo, Ying Liang, Lin Wang, Jing Zhou, Li Yan, Tian-Xin Lin, Shao-Ling Zhang

**Affiliations:** ^1^ Department of Endocrinology, Sun Yat-sen Memorial Hospital, Sun Yat-sen University, Guangzhou, China; ^2^ Department of Pathology, Sun Yat-sen Memorial Hospital, Sun Yat-sen University, Guangzhou, China; ^3^ Department of Urology, Sun Yat-sen Memorial Hospital, Sun Yat-sen University, Guangzhou, China

**Keywords:** adrenocortical carcinoma, adrenocortical adenoma, serum steroid profiling, LC-MS/MS, diagnosis

## Abstract

**Background:**

It is challenging for clinicians to distinguish adrenocortical carcinoma (ACC) from benign adrenocortical adenomas (ACA) in their early stages. This study explored the value of serum steroid profiling as a complementary biomarker for malignancy diagnosis of ACC other than diameter and explored the influence of sex and functional status.

**Methods:**

In this retrospective study, a matched cohort of patients diagnosed with either ACC or ACA based on histopathology was meticulously paired in a 1:1 ratio according to sex, age, and functional status. Eight serum steroids including 11-deoxycortisol, 11-deoxycorticosterone, progesterone, androstenedione, dehydroepiandrosterone (DHEA), dehydroepiandrosterone sulfate (DHEAS), 17-hydroxyprogesterone, and estradiol, were quantified by liquid chromatography tandem mass spectrometry. We conducted a comparative analysis of the clinical characteristics and serum steroid profiles of patients with ACC and ACA, with further subgroup analysis.

**Results:**

The study included 31 patients with ACC and 31 matched patients with ACA. Patients with ACC exhibited significantly larger tumor diameters, lower body mass index (BMI), and higher levels of 11-deoxycortisol, progesterone, and androstenedione than those with ACA. 11-deoxycortisol was the only valuable index for discriminating ACC from ACA, regardless of functional status and sex. Progesterone, DHEA, and DHEAS levels were higher in the functional ACC group than in the non-functional ACC group. Female ACC patients, especially in postmenopausal female exhibited higher levels of androstenedione than male patients. The area under the curve of tumor diameter, 11-deoxycortisol, and BMI was 0.947 (95% CI 0.889–1.000), with a sensitivity of 96.8% and specificity of 90.3%.

**Conclusion:**

Serum steroid profiling serves as a helpful discriminative marker for ACC and ACA, with 11-deoxycortisol being the most valuable marker. For other steroid hormones, consideration of sex differences and functional status is crucial.

## Introduction

Adrenocortical carcinoma (ACC) is a rare cancer with a new case of an estimated 0.5–2 per million per year. Despite advances in both medical and surgical care, it remains a cancer with a poor prognosis, with an average 5-year survival rate of 20%–25%. Prognosis worsens with increasing disease stage (1). Unfortunately, ACC is frequently diagnosed in advanced stages, and treatment options are limited. Hence, early diagnosis of ACC, especially at the localized stage, can be life-saving, with complete surgical removal of the tumor. Therefore, there is an urgent need to identify novel and reliable diagnostic biomarkers of malignancy before surgery is urgent (2). Nearly 40%–60% of ACC is hormonally active, secreting cortisol, sex hormones, and aldosterone, the so-called functional. In contrast, non-functional ACC cases are often overlooked, leading to delayed surgery and rapid progression to distant metastasis (3).

Currently, there are no imaging techniques, hormonal tests, or immunohistochemical markers that can definitively confirm the diagnosis of ACC. Pathology is the gold standard for diagnosing ACC (4). However, adrenal biopsy before surgery is invasive and cannot be performed in patients with poor condition (5), which is not recommended in the routine diagnostic work-up by the guidelines (6). Adrenal tumors with a Weiss score of three falls into a borderline gray area of malignancy. The Weiss score cannot distinguish between benign and malignant tumors and is misdiagnosed in 9%–13% of cases (7). Before surgery, the current guidelines for the diagnostic workup of adrenal tumors recommend imaging and biochemical testing for hormone excess (6). Unenhanced computed tomography (CT) is the imaging method of choice with tumor tissue attenuation of less than 10, indicating the absence of malignancy, with high sensitivity but low specificity (8, 9). Imaging features cannot be used to assess hormonal functionality. Autonomous steroid secretion is a common feature of ACC, with increased release of steroid precursors (10). Therefore, clinical practice guidelines emphasize the importance of steroid precursors, particularly in suspected ACC (6). In recent years, several single-center cohort studies have demonstrated that six to 11 types of plasma steroids can predict ACC (11–14). Some ACC patients showed increased levels of steroid markers (including androstenedione, DHEAS, 11-deoxycortisol, progesterone, 17-hydroxypregnenolone, 17-hydroxyprogesterone [17OHP], and 11-deoxycorticosterone), whereas others did not demonstrate this trend. The ACC is highly heterogeneous. Existing studies have not controlled for important variables such as age, sex, and functional status simultaneously, which may affect the level of steroids and their precursors (11–14).

In this retrospective study, we compared serum steroid profiling and clinical characteristics between patients with ACC and 1:1 ACA control patients, individually matched for sex, age, and functional status from a single medical center in China to identify the most sensitive markers in ACC patients even under stratification analysis.

## Materials and methods

### Patients

This single-center, case-controlled study was conducted at Sun Yat-sen Memorial Hospital, Sun Yat-sen University (Guangzhou, China). Between January 2010 and June 2022, 1,184 patients admitted for the pathological diagnosis of adrenal cortical tumors were enrolled in this study (15).

ACC was diagnosed according to the Weiss score, which comprised nine histological criteria: (i) high nuclear grade; (ii) mitotic rate greater than 5 per 50 high-power fields (HPF); (iii) atypical mitotic figures; (iv) eosinophilic tumor cell cytoplasm (greater than 75% of tumor cells); (v) diffuse architecture (greater than 33% of the tumor); (vi) necrosis; (vii) venous invasion; (viii) sinusoidal invasion; and (ix) capsular invasion. A tumor was labeled as malignant when it met three or more of these histological criteria (4). Tumor staging at diagnosis was based on imaging studies and findings during surgery and pathological examination. The ENSAT staging system consists of stages I (T1N0M0), II (T2N0M0), III (T1–2N1M0 or T3–4N0–1M0), and IV (TanyNanyM1, metastatic ACC) (16). The Ki-67 index was evaluated by immunohistochemistry, which detected the Ki-67 antigen in neoplastic cell populations, indicating cell proliferation. All histological diagnoses were confirmed by the pathologists.

**Figure 1:**
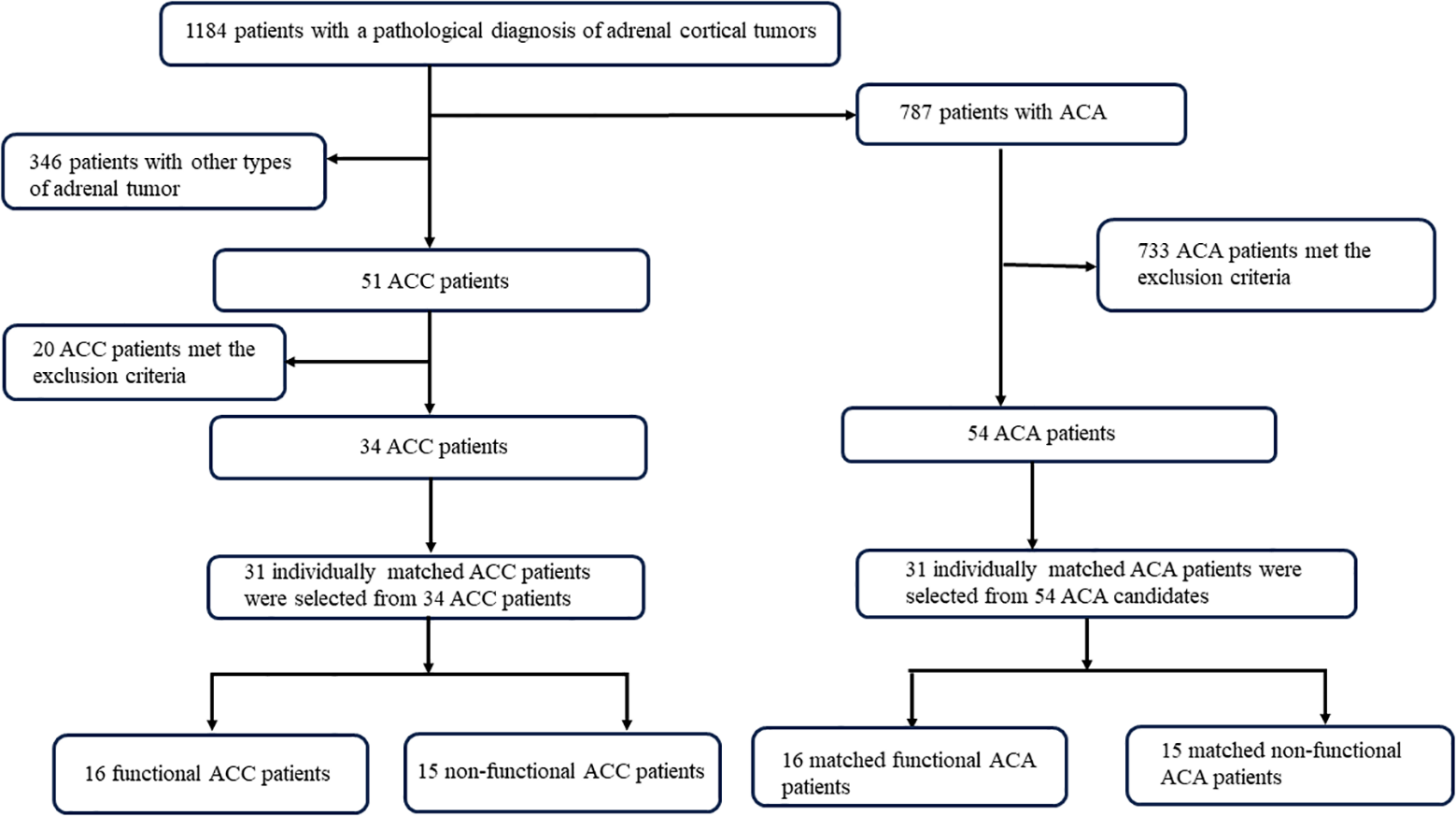
Flowchart of selection process of patients with ACC and ACA. ACC, adrenocortical carcinoma; ACA, adrenocortical adenoma.

Clinical or laboratory evidence of associated conditions was also excluded from this study: (1) lack of preoperative serum samples; (2) patients who took drugs known to alter steroid synthesis or metabolism (e.g., mitotane, ketoconazole, hydrocortisone, mifepristone, etc.) before diagnosis (<6 months); (3) other types of adrenal tumors, such as adrenal ectopia, adrenal cysts, and adrenal pseudocysts confirmed by pathological information; (4) renal insufficiency, estimated glomerular filtration rate ≤60 ml/(min-per 1.73 m^2^); (5) liver insufficiency with transaminases elevated to more than three times the normal value; (6) pregnant women, lactating patients; (7) patients with malignant tumors other than ACC. A total of 346 patients with other types of adrenal tumors were excluded. Of the 51 ACC patients, 17 patients who lacked serum samples and three patients who took drugs known to alter steroid synthesis or metabolism were excluded. In the control group, patients with ACA were referred to our center during the same period. Among the 787 ACA patients, 649 patients who lacked serum samples, 78 patients who had liver or kidney insufficiency, and 60 patients who had other malignant tumors were excluded. A total of 34 ACC patients and 54 ACA patients were enrolled in this study (**Figure 1**). Then, according to age ( ± 5 years), sex, and functional status, we selected 31 ACC patients and 31 matched ACA patients.

The study protocol conformed to the ethical guidelines of the 1975 Declaration of Helsinki by the Ethics Committee of Sun Yat-sen University, and written informed consent was obtained from all study participants.

### Definition of hormone excess

The definition of hormone excess was evaluated after a clinical workup at our center. Cortisol excess was determined in patients with signs or symptoms of excess hormones, increased 24-hour urinary free cortisol, and high plasma cortisol, which could not be suppressed with overnight dexamethasone at a dose of 1 mg (17). Cortisol and urinary-free cortisol levels were measured by chemiluminescence immunoassay (CLIA) using commercial kits (IMMULITE2000 Cortisol, UK). Excess aldosterone was defined as failure to suppress post-infusion aldosterone levels to 10 ng/dl with an elevated aldosterone-to-renin ratio (ARR) ≥25 ng/dl per ng/ml per h (18). Aldosterone and plasma renin activities were measured by CLIA using commercial kits (Snibe MAGLUMI800, China). The diagnosis of androgen excess requires signs and symptoms in women, including hirsutism, acne, seborrhea, androgenic alopecia, and disappearance of symptoms after surgery. Meanwhile, free testosterone was more than 2.64 nmol/l in female. Estrogen secreting was defined as serum levels of estradiol higher than 442.62 nmol/l before surgery, accompanied by loss of hyposexuality, erectile dysfunction, and gynecomastia and normalized after surgery (19, 20). Androgen and estrogen levels were measured by CLIA, using commercial kits (Snibe MAGLUMI800, China). Tumors with no evidence of hormone secretion or the patient had none of the above signs and symptoms were considered inactive.

### Measurements of steroid metabolites by LC–MS/MS

Blood samples were collected at 08:00 A.M. Serum was separated and stored at −80°C until assay. Samples were shipped on dry ice to the KINGMED DIAGNOSTICS, where steroids were measured by LC–MS/MS according to an established method. Steroid analysis of peripheral venous serum was accomplished by LC–MS/MS with simultaneous measurement of eight steroids (11-deoxycortisol, 11-deoxycorticosterone, progesterone, androstenedione, DHEA, DHEAS, 17-OHP, and estradiol).

Serum 11-deoxycorticosterone, progesterone, DHEA, and DHEAS were analyzed using SHIMADZU LC-MS/MS 8060. The calibration range was 0.03 nmol/l–6.80 nmol/l, 0.32 nmol/l–160 nmol/l, 11.4 nmol/l–374 nmol/l, and 0.03 nmol/l–13.6 μmol/l, respectively. The lowest limit of quantification was 0.03 nmol/l, 0.32 nmol/l, 11.4 nmol/l, and 0.03 μmol/l, respectively. The intra- and inter-assay coefficients of variation for the 4 steroid hormones were <5%. Serum androstenedione, 11-deoxycortisol, and 17-hydroxyprogesterone levels were analyzed using an AB Sciex Triple Quad 5500. The calibration range was 0.44 nmol/l–43.7 nmol/l, 0.10 nmol/l–30.0 nmol/l, and 0.10 nmol/l–30.0 nmol/l, respectively. The lowest limit of quantification was 0.44 nmol/l, 0.10 nmol/l, and 0.50 nmol/l, respectively. The intra- and interassay coefficients of variation for the three steroid hormones were <5%. Serum estradiol was analyzed by Thermo TSQ Altis. The calibration range was 3.71 pmol/l–3,680 pmol/l. The lowest limit of quantification is 3.71 pmol/l. The intra- and interassay coefficients of variation were <7.5%. At the same time, to ensure the accuracy of the testing results of the program, we regularly participated in the External Quality Assessment (EQA) program organized by the Royal College of Pathologists of Australia (RCPA) and the inter-room quality evaluation activities of the Clinical Laboratory Center of the National Health and Health Commission of China.

### Biochemical measurements

Biochemical parameters, creatinine, alanine transaminase (ALT), aspartate transaminase (AST), serum potassium, sodium, chlorine, fasting glucose, albumin, alpha-fetoprotein (AFP), and carcinoembryonic antigen (CEA) were measured using a standardized and certified program with an automatic biochemical analyzer (AU5800, Beckman Coulter, USA) at Sun Yat-sen Memorial Hospital.

### Statistical analysis

Baseline continuous data are expressed as mean ± SD or median (interquartile range) for normally or non-normally distributed data, respectively. Categorical data are presented as numbers (percentages). Differences in these characteristics between the ACC and ACA groups were compared using the paired samples t-test or Wilcoxon test for continuous variables and χ^2^ tests for categorical variables. Subgroup analysis stratified by sex (female or male) and functional status (functional or non-functional) was also performed. Univariate logistic analysis with odds ratios (ORs) and 95% confidence intervals (95% CIs) was used to investigate the risk factors for ACC. Receiver operating characteristic (ROC) analysis was used for steroid hormone profiling and clinical characteristics to distinguish ACC from ACA. Based on the highest Youden index, the cutoff value, sensitivity, specificity, positive predictive value (PPV), and negative predictive value (NPV) were calculated. A nomogram was constructed to visualize the results of the multivariate analysis. Surv_cutpoint (“survminer,” R package) was used to separate the plasma metabolites into two groups. All statistical analyses were conducted using SPSS 26 (SPSS Inc., Chicago, IL, USA) and Medcalc 20.218, with a two-tailed P <0.05 considered statistically significant.

## Results

A total of 1,184 patients with a pathological diagnosis of adrenal cortical tumors were recruited. Of these, 51 were diagnosed with ACC, resulting in a cohort prevalence of 4.3%.

### Comparisons of clinical characteristics between patients with ACC and ACA

The baseline characteristics of the 34 ACC and 54 ACA patients are summarized (**Table 1**). As expected, ACC patients had significantly higher tumor diameters, BMI, serum potassium levels, and Weiss scores (*P <*0.05). For steroid profiling, only progesterone was significantly higher in ACC patients than in ACA patients (0.32 [0.16–0.73] vs. 0.16 [0.08–0.33] nmol/l).

**Table 1:** Demographic and clinical characteristics of the ACC and ACA patients.

Characteristics	ACC (n = 34)	ACA (n = 54)	*P-*value
Demographic characteristics			
Sex, male/female	11/23	19/35	NS
Age, y	49.50 ± 14.54	44.06 ± 13.00	0.085
BMI, kg/m^2^	23.10 ± 2.86	24.66 ± 3.58	0.011
SBP, mmHg	131.82 ± 23.30	138.87 ± 20.93	0.117
DBP, mmHg	81.82 ± 15.65	87.80 ± 15.60	0.104
Serum biochemical characteristics			
Creatinine	69.15 ± 13.04	74.94 ± 16.94	0.119
AST, U/l	22.32 ± 11.15	20.15 ± 8.31	0.545
ALT, U/l	26.00 ± 20.85	23.13 ± 11.77	0.659
Fasting glucose, mmol/l	5.61 ± 1.62	5.27 ± 1.10	0.654
Serum potassium, mmol/l	3.41 ± 0.63	3.78 ± 0.36	0.007
Serum sodium, mmol/l	140.79 ± 0.63	140.37 ± 2.70	0.137
Serum chlorine, mmol/l	104.65 ± 3.25	105.57 ± 2.47	0.241
Albumin, g/l	37.22 ± 4.05	39.08 ± 4.66	0.075
AFP, ng/ml	2.76 (1.84–4.43)	2.73 (1.69–4.12)	0.519
CEA, ng/ml	2.10 (1.60–5.00)	2.20 (1.45–3.05)	0.313
LDH, U/l	238.00 (160.00–401.00)	190.00 (157.75–250.50)	0.142
Tumor diameter, mm	76.85 ± 31.22	32.04 ± 16.35	<0.001
Tumor present at sampling			
Primary tumor	23 (67.7%)	54	
Primary tumor + metastases	3 (8.8%)		
Local recurrence	1 (2.9%)		
Distant recurrence	5 (14.7%)		
Local + distant recurrence	2 (5.9%)		
ENSAT stage at initial diagnosis			
1	3 (8.8%)		
2	9 (24.5%)		
3	11 (32.4%)		
4	11 (32.4%)		
Weiss score	5.00 (4.00–6.00)	0	
Ki-67% index	15.00 (9.00–27.50)	2.00 (1.00–3.00)	<0.001
Resection status			
R0	26 (83.9%)		
R1	0 (0.00%)		
R2	2 (6.4%)		
Rx	3 (9.7%)		
Hormone secretion			
Functional	18 (53.0%)	30 (55.6%)	NS
cortisol excess	11	30	
cortisol and aldosterone excess	2		
cortisol and androgen excess	1		
cortisol, aldosterone and androgen excess	2		
aldosterone and androgen excess	1		
aldosterone excess	1		
Non-functional	16 (46.0%)	24 (44.4%)	NS
Steroid hormone profiling			
DHEA, nmol/l	4.36 (1.17–9.16)	3.39 (1.82–7.74)	0.776
DHEAS, μmol/l	1.31 (0.72–3.31)	0.97 (0.35–2.38)	0.169
Androstenedione, nmol/l	2.32 (1.39–4.59)	2.39 (1.21–3.79)	0.411
11-deoxycorticosterone, nmol/l	0.18 (0.11–0.44)	0.18 (0.10–0.37)	0.628
Aldosterone, ng/l	127.00 (89.65–283.45)	169.30 (128.00–271.00)	0.270
11-deoxycortisol, nmol/l	3.00 (1.04–6.92)	2.00 (1.04–3.62)	0.166
Cortisol, nmol/l	450.08 (329.10–811.53)	488.53 (349.71–741.92)	0.877
Progesterone, nmol/l	0.32 (0.16–0.73)	0.16 (0.08–0.33)	0.023
17-OHP, nmol/l	1.84 (0.87–4.49)	1.75 (0.95–3.49)	0.915
Estradiol, pmol/l	70.40 (19.20–127.60)	60.55 (20.70–158.75)	0.781

Values are presented as mean ± SD or median (25^th^–75^th^ quartiles) for continuous variables and n (%) for categorical variables.

ACC, adrenocortical carcinoma; ACA, adrenocortical adenoma; DHEA, dehydroepiandrosterone; DHEAS, dehydroepiandrosterone-sulfate;17-OHP, 17-hydroxyprogesterone; ALT, alanine transaminase; AST, aspartate transaminase; BMI, body mass index; DBP, diastolic blood pressure; SBP, systolic blood pressure; CT, computerized tomography; AFP, Alpha-fetoproteinl; CEA, Carcinoembryonic antigen; LDH, lactate dehydrogenase; NS, non-significant.

The 31 patients with ACA were selected after careful matching with similar age ( ± 5 years), and sex, and functional status. Finally, 31 ACC patients (mean age, 48.55 ± 14.28; 67.7% female; 51.6% functional tumor) and 31 matched ACA controls (mean age, 48.81 ± 10.43; 67.7% female; 51.6% functional tumor) were included. As matched beforehand, there were no significant differences in age, sex, and functional status between ACC and ACA patients.

**Figure 2:**
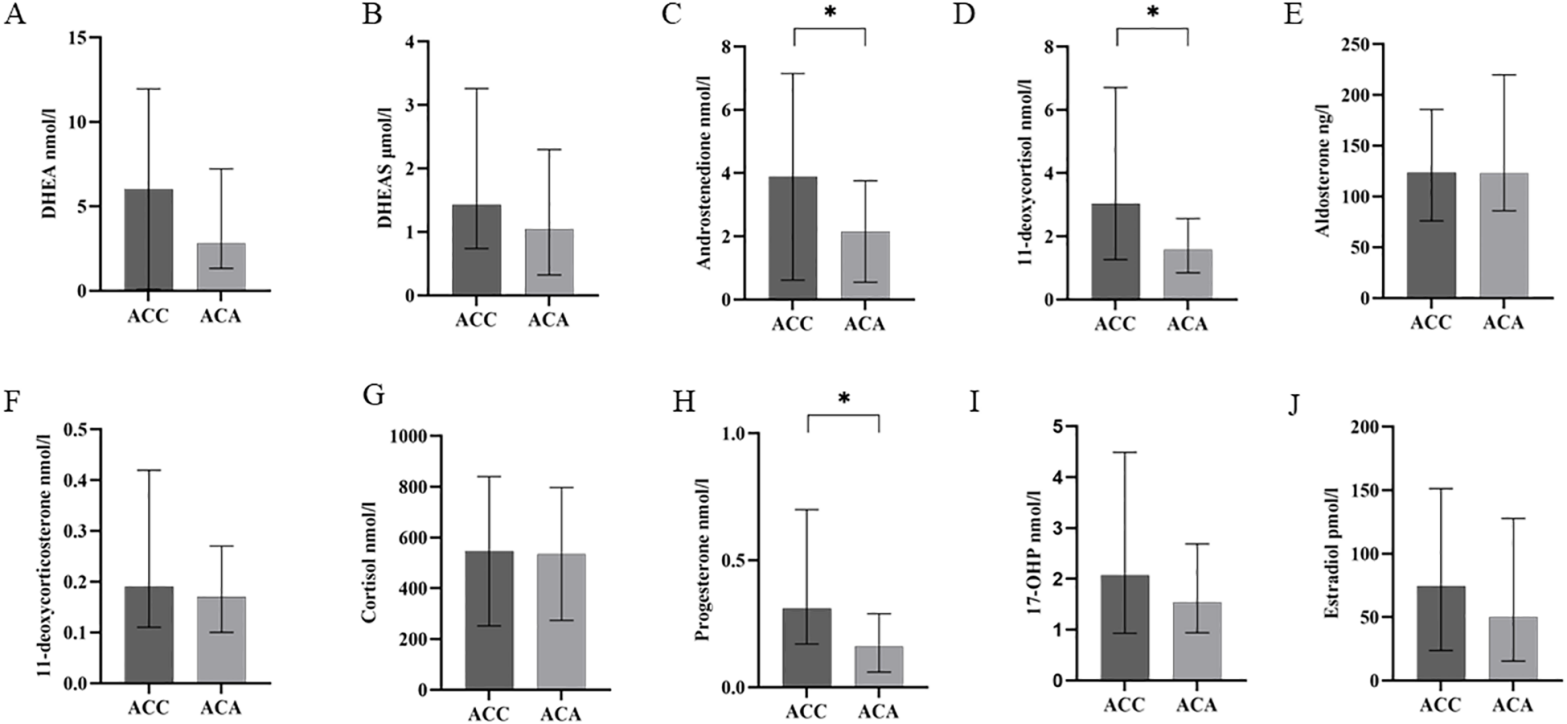
Steroid profiling in patients with ACC and ACA. Quantity of the eight steroid hormones [DHEA **(A)**, DHEAS **(B)**, androstenedione **(C)**, 11-deoxycortisol **(D)**, aldosterone **(E)**, 11-deoxycorticosterone **(F)**, cortisol **(G)**, progesterone **(H)**, 17-OHP **(I)**, estradiol **(J)**] as measured by LC–MS/MS in patients with ACC and ACA. *P less than 0.05, higher than ACA. 17-OHP, 17-hydroxyprogesterone; DHEA, dehydroepiandrosterone; DHEAS, dehydroepiandrosterone-sulfate; ACC, adrenocortical carcinoma; ACA, adrenocortical adenoma; LC-MS/MS, liquid chromatography tandem mass spectrometry.

The clinical and biomedical characteristics of the patients with 31 ACC and 31 ACA patients were shown in **Table 2**. Among the 31 ACC patients, 16 had suffered autonomous hypersecretion of hormones. Nine ACC patients had autonomous cortisol hypersecretion. Two patients had co- secretions of cortisol and aldosterone. One patient had a co-secretion of cortisol and androgen. Two patients had co- secretions of cortisol, aldosterone, and androgen. One patient showed autonomous aldosterone hypersecretion. One patient had co-secretion of aldosterone and androgen. Of the 31 patients with ACA, 16 showed only autonomous cortisol hypersecretion. A total of 24 ACC patients underwent R0 resection, two underwent R2 resection, and three ACC patients underwent RX resection. In ACC cases, 71.0% of patients had localized disease (ENSAT stages I–III). The median Weiss score of ACC patients was 5. As expected, ACC patients had significantly higher diameters of adrenal masses (76.22 mm ± 32.30 mm vs. 32.19 mm ± 18.98 mm, *P <*0.001). Additionally, ACC patients had lower levels of BMI (22.88 kg/m^2^ ± 2.73 kg/m^2^ vs. 25.43 kg/m^2^ ± 2.88 kg/m^2^, *P <*0.001) than ACA controls. The concentrations of steroid hormones in ACC and ACA patients are presented (**Table 2**, **Figure 2**). Overall, ACC patients exhibited significantly higher concentrations of hormones than ACA patients, including 11-deoxycortisol (3.03 [1.26–6.71] vs. 1.58 [0.85–2.56] nmol/l, *P <*0.001), progesterone (0.31 [0.17–0.70] vs. 0.16 [0.06–0.29] nmol/l, *P* = 0.022), and androstenedione (2.52 [1.48–4.70] vs. 1.76 [1.01–2.75] nmol/l, *P* = 0.001). The largest difference was observed for 11-deoxycortisol, which in patients with ACC was 1.93-fold higher than in those with ACA. Notably, end products such as cortisol and aldosterone were not found to be statistically different between the two groups. No significant differences were found in the levels of DHEA, DHEAS, 11-deoxycorticosterone, 17-OHP, and estradiol between patients with ACC and ACA. In addition, there were no statistical differences in systolic blood pressure (SBP), diastolic blood pressure (DBP), serum creatinine, serum sodium, serum potassium, serum chloride, albumin, ALT, AST, fasting glucose, AFP, and CEA levels between patients with ACC and ACA.

**Table 2:** Demographic and clinical characteristics of the ACC and ACA patients.

Characteristics	ACC (n = 31)	ACA (n = 31)	*P-*value
Demographic characteristics			
Sex, male/female	10/21	10/21	NS
Age, y	48.55 ± 14.28	48.81 ± 10.43	0.859
BMI, kg/m^2^	22.88 ± 2.73	25.43 ± 2.88	<0.001
SBP, mmHg	130.54 ± 23.61	138.68 ± 17.08	0.182
DBP, mmHg	81.52 ± 16.16	86.03 ± 14.14	0.285
Serum biochemical characteristics			
Creatinine	70.32 ± 12.87	75.87 ± 17.04	0.102
AST, U/l	22.32 ± 11.65	20.61 ± 8.85	0.571
ALT, U/l	26.35 ± 21.77	24.71 ± 13.18	0.888
Fasting glucose, mmol/l	5.31 ± 1.29	5.31 ± 0.83	0.854
Serum potassium, mmol/l	3.47 ± 0.60	3.72 ± 0.36	0.081
Serum sodium, mmol/l	140.59 ± 2.52	140.65 ± 2.81	0.853
Serum chlorine, mmol/l	105.02 ± 2.72	105.90 ± 2.72	0.194
Albumin, g/l	37.18 ± 4.02	38.66 ± 4.11	0.082
AFP, ng/ml	3.38 ± 1.73	3.06 ± 1.46	0.427
CEA, ng/ml	2.15 (1.89–4.75)	2.25 (1.57–2.78)	0.836
LDH, U/l	228.00 (151.75–369.00)	188.00 (159.00–286.00)	0.153
Tumor diameter, mm	76.22 ± 32.30	32.19 ± 18.98	<0.001
Tumor present at sampling			
Primary tumor	22 (71.0%)	31 (100.0%)	
Primary tumor + metastases	2 (6.5%)		
Local recurrence	1 (3.1%)		
Distant recurrence	4 (12.9%)		
Local + distant recurrence	2 (6.5%)		
ENSAT stage at initial diagnosis			
1	3 (9.7%)		
2	8 (25.8%)		
3	11 (35.4%)		
4	9 (29.0%)		
Weiss score	5.00 (4.00–6.00)		
Ki-67% index	15.00 (8.00–25.00)		
Hormone secretion			
Functional	16 (51.6%)	16 (51.6%)	
cortisol excess	9	16	
cortisol and aldosterone excess	2		
cortisol and androgen excess	1		
cortisol, aldosterone and androgen excess	2		
aldosterone and androgen excess	1		
aldosterone excess	1		
Non-functional	15 (48.4%)	15 (48.4%)	
Steroid hormone profiling			
DHEA, nmol/l	4.36 (1.63–9.07)	2.82 (1.32–7.22)	0.468
DHEAS, μmol/l	1.43 (0.74–3.26)	1.04 (0.32–2.30)	0.290
Androstenedione, nmol/l	2.52 (1.48–4.70)	1.76 (1.01–2.75)	0.001
11-deoxycorticosterone, nmol/l	0.19 (0.11–0.42)	0.17 (0.10–0.27)	0.098
Aldosterone, ng/l	105.70 (67.07–773.55)	153.00 (116.60–266.80)	0.984
11-deoxycortisol, nmol/l	3.03 (1.26–6.71)	1.58 (0.85–2.56)	<0.001
Cortisol, nmol/l	449.39 (334.02–773.55)	460.77 (307.43–738.27)	0.082
Progesterone, nmol/l	0.31 (0.17–0.70)	0.16 (0.06–0.29)	0.022
17-OHP, nmol/l	2.07 (0.93–4.49)	1.54 (0.94–2.69)	0.357
Estradiol, pmol/l	74.40 (23.80–151.30)	50.30 (15.40–127.70)	0.814

Values are presented as mean ± SD or median (25th–75th quartiles) for continuous variables and n (%) for categorical variables.

ACC, adrenocortical carcinoma; ACA, adrenocortical adenoma; DHEA, dehydroepiandrosterone; DHEAS, dehydroepiandrosterone-sulfate;17-OHP, 17-hydroxyprogesterone; ALT, alanine transaminase; AST, aspartate transaminase; BMI, body mass index; DBP, diastolic blood pressure; SBP, systolic blood pressure; CT, computerized tomography; AFP, Alpha-fetoprotein; CEA, Carcinoembryonic antigen; LDH, lactate dehydrogenase; NS, non-significant.

### Subgroup analysis based on functional status and sex

Subsequently, a separate comparison was made between patients with functional ACC (n = 16) and those with functional ACA (n = 16). As shown (**Table 3**), the concentrations of DHEA (4.31 [1.83–9.88] vs. 1.53 [0.80–3.37] nmol/l, *P* = 0.017), DHEAS (1.57 [0.79–4.42] vs. 0.34 [0.19–1.66] μmol/l, *P* = 0.030), androstenedione (3.53 [1.50–7.86] vs. 1.45 [0.87–2.65] nmol/l, *P* = 0.010], 11-deoxycortisol (4.02 [3.06–9.47] vs. 2.40 [1.59–4.14] nmol/l, *P* = 0.007], and progesterone (0.25 [0.17–0.36) vs. 0.15 [0.06–3.37] nmol/l, *P* = 0.008] were significantly higher in ACC group. Furthermore, ACC patients had significantly lower BMI (23.05 kg/m^2^ ± 2.54 kg/m^2^ vs. 24.92 kg/m^2^ ± 2.96 kg/m^2^, *P* = 0.013) and larger tumor diameters (78.78 mm ± 37.53 mm vs. 31.00 mm ± 23.22 mm, *P <*0.001) than ACA patients. Non-functional ACC patients (n = 15) had significantly higher concentrations of 11-deoxycortisol (1.41 [0.66–2.96] vs. 0.92 [0.56–1.25] nmol/l, *P* = 0.023), lower BMI (23.02 kg/m^2^ ± 3.01 kg/m^2^ vs. 25.63 kg/m^2^ ± 2.84 kg/m^2^, *P* = 0.003) and larger tumor diameters (73.49 mm ± 26.68 mm vs. 24.99 mm ± 9.27 mm, *P <*0.001) than non-functional ACA patients (n = 15). Additionally, it was observed that the functional ACA group had significantly lower levels of DHEA and DHEAS but higher levels of 11-deoxycortisol and cortisol (all *P <*0.05) than the non-functional ACA group (**Table 3**).

**Table 3:** Comparison of serum steroids between ACC and ACA stratified by functional status.

Variables	Functional group		Non-functional group		*^a^P-*value	*^b^P-*value	*^c^P-*value
	ACC	ACA	ACC	ACA			
N	16	16	15	15	NS	NS	NS
Sex, male/female	5/11	5/11	5/10	5/10	NS	NS	NS
Age	47.94 ± 14.32	47.38 ± 9.74	49.20 ± 14.71	49.00 ± 11.24	0.622	0.396	0.572
BMI, kg/m^2^	23.05 ± 2.54	24.92 ± 2.96	23.02 ± 3.01	25.63 ± 2.84	0.013	0.003	0.800
Tumor diameter, mm	78.78 ± 37.53	31.00 ± 23.22	73.49 ± 26.68	24.99 ± 9.27	<0.001	<0.001	0.030
DHEA, nmol/l	4.31 (1.83–9.88)	1.53 (0.80–3.37)	4.36 (0.69–8.66)	5.41 (2.82–11.98)	0.017	0.125	<0.001
DHEAS, μmol/l	1.57 (0.79–4.42)	0.34 (0.19–1.66)	0.95 (0.62–2.91)	1.77 (0.77–3.04)	0.030	0.281	0.012
Androstenedione, nmol/l	3.53 (1.50–7.86)	1.45 (0.87–2.65)	2.29 (1.40–4.34)	2.09 (1.22–2.90)	0.010	0.078	0.318
11-deoxycorticosterone, nmol/l	0.33 (0.12–1.18)	0.19 (0.11–0.41)	0.15 (0.09–0.34)	0.14 (0.05–0.23)	0.233	0.245	0.101
Aldosterone, ng/l	132.40 (65.39–236.10)	178.80 (94.40–281.13)	96.00 (53.00–123.40)	108.80 (85.90–201.90)	0.796	0.173	0.967
11-deoxycortisol, nmol/l	4.02 (3.06–9.47)	2.40 (1.59–4.14)	1.41 (0.66–2.96)	0.92 (0.56–1.25)	0.007	0.023	0.001
Cortisol, nmol/l	719.25 (393.23–873.85)	739.74 (455.78–902.73)	341.33 (261.15–549.74)	370.36 (294.02–469.63)	0.679	0.496	0.001
Progesterone, nmol/l	0.25 (0.17–0.36)	0.15 (0.06–3.37)	0.43 (0.12–4.81)	0.24 (0.07–1.49)	0.008	0.443	0.151
17-OHP, nmol/l	2.14 (1.03–4.09)	1.25 (0.87–2.08)	1.62 (0.85–4.50)	2.10 (0.96–4.93)	0.070	0.394	0.110
Estradiol, pmol/l	71.15 (27.45–100.13)	40.10 (13.58–111.40)	74.40 (14.70–214.80)	70.90 (15.4–322.80)	0.326	0.820	0.281

Values are presented as mean ± SD or median (25th–75th quartiles) for continuous variables and n (%) for categorical variables.

ACC, adrenocortical carcinoma; ACA, adrenocortical adenoma; DHEA, dehydroepiandrosterone; DHEAS, dehydroepiandrosterone-sulfate;17-OHP, 17-hydroxyprogesterone; BMI, body mass index; NS, non-significant.

^a^P-value refers to comparison of functional ACC and functional ACA.

^b^P-value refers comparison of non-functional ACC and non-functional ACA.

^c^P-value refers comparison of non-functional ACA and functional ACA.

Furthermore, we conducted separate analyses of steroid profiling and clinical features in males and females, as presented in **Table 4**. The concentration of 11-deoxycortisol was significantly elevated in the ACC group in both males (3.09 [1.92–3.96] vs 1.66 [0.81–2.58] nmol/l, *P* = 0.017) and females (2.96 [0.82–12.16] vs 1.58 [0.83–2.95] nmol/l, *P* = 0.005). Specifically, in the female subgroup, the androstenedione level was higher in ACC patients than in ACA patients (3.26 [1.59–4.63] vs. 1.58 [0.83–2.94] nmol/l, *P* = 0.006). Compared to ACA patients, BMI was significantly smaller and tumor diameter was larger in both male and female patients (all *P <*0.05).

**Table 4:** Comparison of serum steroids between function ACC and ACA stratified by gender.

Variables	Female		Male		*^a^P-*value	*^b^P*-value
	ACC	ACA	ACC	ACA		
N	21 (33.9%)	21 (33.9%)	10 (16.1%)	10 (16.1%)	NS	NS
Functional status/non-functional status	11/10	11/10	5/5	5/5		
Age	47.29 ± 12.88	48.00 ± 10.14	51.20 ± 17.30	52.00 ± 10.83	0.917	0.859
BMI, kg/m^2^	22.77 ± 2.83	24.71 ± 2.50	23.58 ± 2.55	26.43 ± 3.38	0.003	0.015
Tumor diameter, mm	69.57 ± 29.80	32.42 ± 16.62	90.20 ± 34.41	31.73 ± 24.22	<0.001	<0.001
DHEA, nmol/l	4.45 (2.53–7.32)	2.51 (1.53–6.69)	2.31 (0.62–13.36)	3.48 (0.97–7.48)	0.689	0.508
DHEAS, μmol/l	1.51 (0.71–3.58)	0.69 (0.25–2.46)	0.95 (0.66–3.09)	1.34 (0.86–2.12)	0.357	0.575
Androstenedione, nmol/l	3.26 (1.59–4.63)	1.58 (0.83–2.94)	2.08 (1.32–6.02)	1.82 (1.06–2.63)	0.006	0.103
11-deoxycorticosterone, nmol/l	0.19 (0.10–0.54)	0.17 (0.09–0.26)	0.20 (0.13–0.41)	0.13 (0.09–0.40)	0.112	0.575
Aldosterone, ng/l	123.25 (74.38–185.75)	169.30 (111.00–247.60)	79.55 (45.58–121.63)	130.45 (112.20–315.63)	0.433	0.074
11-deoxycortisol, nmol/l	2.96 (0.82–12.16)	1.58 (0.83–2.95)	3.09 (1.92–3.96)	1.66 (0.81–2.58)	0.005	0.017
Cortisol, nmol/l	415.14 (337.70–813.26)	469.66 (370.63–816.27)	450.08 (259.52–614.33)	365.78 (291.56–592.65)	0.903	0.721
Progesterone, nmol/l	0.35 (0.20–1.29)	0.24 (0.07–0.61)	0.20 (0.82–0.40)	0.16 (0.06–0.19)	0.140	0.074
17-OHP, nmol/l	2.07 (0.91–4.35)	1.05 (0.82–2.67)	1.92 (0.91–4.65)	1.66 (1.47–3.48)	0.192	0.959
Estradiol, pmol/l	90.00 (31.10–206.55)	20.80 (11.70–248.45)	54.35 (17.54–76.70)	53.80 (40.10–79.22)	0.434	0.333

Values are presented as mean ± SD or median (25th–75th quartiles) for continuous variables and n (%) for categorical variables.

ACC, adrenocortical carcinoma; ACA, adrenocortical adenoma; DHEA, dehydroepiandrosterone; DHEAS, dehydroepiandrosterone-sulfate;17-OHP, 17-hydroxyprogesterone; BMI, body mass index; NS, non-significant.

^a^P-value refers comparison of female ACC and female ACA.

^b^P-value refers comparison of male ACC and male ACA.

Finally, we conducted separate analyses of steroid profiles and clinical features based on menopausal status in female patients, as presented in **Table 5**. The concentrations of androstenedione (2.27 [1.46–4.59] vs 1.35 [0.85–2.15] nmol/l, *P* = 0.045) and 11-deoxycortisol (6.50 [1.71–24.65] vs 1.34 [0.84–2.54] nmol/l, *P* = 0.049) were significantly elevated in the post-menopausal ACC female patient group, but not in the pre-menopausal female group.

**Table 5:** Comparison of serum steroids between ACC and ACA stratified by menopausal status in females.

Variables	Post-menopausal		Pre-menopausal		*^a^P-*value	*^b^P*-value
	ACC	ACA	ACC	ACA		
N	10	10	11	11	NS	NS
Functional status/non-functional status	6/4	6/4	5/6	5/6		
Age	54.00 ± 9.26	54.00 ± 5.25	39.00 ± 9.06	43.00 ± 7.83	0.971	0.847
BMI, kg/m^2^	23.99 ± 3.07	25.59 ± 2.11	21.67 ± 2.17	23.90 ± 2.65	0.190	0.023
Tumor diameter, mm	72.00 ± 33.24	28.23 ± 9.26	67.36 ± 27.76	36.23 ± 21.02	<0.001	0.005
DHEA, nmol/l	3.21 (0.59–4.67)	3.52 (1.28–8.35)	5.98 (4.36–13.23)	2.29 (1.75–5.82)	0.393	0.123
DHEAS, μmol/l	1.08 (0.41–2.05)	0.54 (0.22–2.49)	2.12 (0.95–4.59)	0.77 (0.27–2.62)	0.684	0.094
Androstenedione, nmol/l	2.27 (1.46–4.59)	1.35 (0.85–2.15)	3.36 (1.60–6.54)	2.90 (1.22–4.75)	0.045	0.250
11-deoxycorticosterone, nmol/l	0.24 (0.72–1.99)	0.15 (0.07–0.24)	0.19 (0.11–0.37)	0.23 (0.10–0.27)	0.449	0.948
Aldosterone, ng/l	144.25 (76.18–395.38)	123.35 (66.70–231.55)	127.15 (93.28–203.70)	128.00 (92.00–156.00)	0.829	0.888
11-deoxycortisol, nmol/l	6.50 (1.71–24.65)	1.34 (0.84–2.54)	2.50 (0.66–6.71)	1.77 (0.60–4.30)	0.049	0.470
Cortisol, nmol/l	467.84 (336.26–850.21)	371.33 (281.56–565.12)	415.14 (338.40–773.55)	735.21 (469.63–921.79)	0.290	0.158
Progesterone, nmol/l	0.34 (0.18–0.48)	0.17 (0.05–0.42)	0.36 (0.17–6.72)	0.24 (0.08–1.49)	0.290	0.340
17-OHP, nmol/l	2.70 (0.94–4.96)	0.95 (0.66–2.24)	1.49 (0.50–4.49)	1.55 (0.87–4.93)	0.112	0.743
Estradiol, pmol/l	59.05 (9.53–266.38)	11.70 (5.77–55.85)	90.00 (66.4–198.30)	127.70 (20.80–440.50)	0.226	0.922

Values are presented as mean ± SD or median (25th–75th quartiles) for continuous variables and n (%) for categorical variables.

ACC, adrenocortical carcinoma; ACA, adrenocortical adenoma; DHEA, dehydroepiandrosterone; DHEAS, dehydroepiandrosterone-sulfate;17-OHP, 17-hydroxyprogesterone; BMI, body mass index; NS, non-significant.

^a^P-value refers comparison of post-menopausal female ACC and post-menopausal female ACA.

^b^P-value refers comparison of pre-menopausal female ACC and pre-menopausal female ACA.

A Ki-67% index ≥20% is considered as one of the poor prognostic factors in ACC (21); therefore, we divided the patients into two groups based on the Ki-67% index (**Table 6**). In the Ki-67% ≥20% group, only 17-OHP was higher.

**Table 6:** Demographic and clinical characteristics of the ACC patients based on Ki-67% index.

Characteristics	Ki-67% ≥20 (n = 14)	Ki-67% <20 (n = 17)	*P-*value
Demographic characteristics			
Age, y	48.00 ± 15.48	48.00 ± 13.58	0.721
Weiss score	5.00 (3.75–6.00)	4.00 (3.50–5.00)	0.230
Ki-67% index	27.50 (20.00–41.25)	10.00 (6.50–13.50)	<0.001
Tumor diameter, mm	84.00 (64.75–101.25)	62.00 (43.70–81.50)	0.100
Steroid hormone profiling			
DHEA, nmol/l	4.31 (1.38–10.78)	4.36 (1.56–7.32)	0.953
DHEAS, μmol/l	1.58 (0.85–3.31)	1.19 (0.57–3.41)	0.518
Androstenedione, nmol/l	4.02 (1.55–6.01)	2.19 (1.39–3.37)	0.200
11-deoxycorticosterone, nmol/l	0.33 (0.14–0.78)	0.12 (0.09–0.35)	0.128
Aldosterone, ng/l	139.25 (52.05–285.45)	118.35 (76.03–149.20)	0.507
11-deoxycortisol, nmol/l	3.55 (2.39–10.24)	2.19 (0.82–5.46)	0.128
Cortisol, nmol/l	500.25 (378.15–819.51)	341.33 (277.24–753.41)	0.279
Progesterone, nmol/l	0.35 (0.21–0.81)	0.24 (0.09–0.72)	0.279
17-OHP, nmol/l	3.63 (1.18–6.77)	1.45 (0.89–3.36)	0.040
Estradiol, pmol/l	71.15 (23.03–120.18)	74.40 (24.75–174.80)	1.000

Values are presented as mean ± SD or median (25th–75th quartiles) for continuous variables and n (%) for categorical variables.

DHEA, dehydroepiandrosterone; DHEAS, dehydroepiandrosterone-sulfate;17-OHP, 17-hydroxyprogesterone.

### The value and cut-off of steroid profiling for ACC screening

Steroids measured by LC-MS/MS are shown in **Table 7**. The most appropriate cutoff values were calculated using Youden statistics. DHEAS had the highest sensitivity (87.1%) and specificity (35.5%) with a cutoff value of 0.50 μmol/l, an area under the curve (AUC) of 0.599. Androstenedione had a sensitivity of 48.4% and the highest specificity of 87.1% with a cutoff value of 3.01 nmol/L, an area under the curve (AUC) of 0.681.

**Table 7:** Receiver operating characteristics (ROC) for steroid profiling for detecting ACC.

	AUC (95% CI)	Cut-off Value	Sensitivity (95% CI)	Specificity (95% CI)
11-deoxycortisol, nmol/l	0.689 (0.556–0.823)	1.99	71.0 (52.0–85.8)	64.5 (45.4–80.8)
Androstenedione, nmol/l	0.681 (0.548–0.814)	3.01	48.4 (30.2–66.9)	87.1 (70.2–96.4)
DHEA, nmol/l	0.684 (0.383–0.679)	2.82	64.5 (45.4–80.8)	51.6 (33.1–69.8)
Progesterone, nmol/l	0.640 (0.498–0.782)	0.16	77.4 (58.9–90.4)	51.6 (33.1–69.8)
11-deoxycorticosterone, nmol/l	0.573 (0.428–0.719)	0.30	42.0 (24.5–60.9)	80.7 (62.5–92.5)
17-OHP, nmol/l	0.566 (0.396–0.691)	2.69	45.2 (27.3–64.0)	77.4 (58.9–90.4)
Estradiol, pmol/l	0.553 (0.406–0.700)	62.5	58.1 (39.1–75.5)	61.3 (42.2–78.2)
DHEAS, μmol/l	0.599 (0.456–0.743)	0.50	87.1 (70.2–96.4)	35.5 (19.2–54.6)

AUC, area under the curve; ACC, adrenocortical carcinoma; DHEA, dehydroepiandrosterone; DHEAS, dehydroepiandrosterone-sulfate;17-OHP, 17-hydroxyprogesterone.

### Diagnostic test performance for ACC

Univariate logistic regression analysis was used to identify potential risk factors for ACC. Univariate analysis revealed that BMI (OR 0.738; 95% CI [0.594–0.918]; *P* = 0.006), tumor diameter (OR 1.079, 95% CI [1.038–1.121]; *P* < 0.001), 11-deoxycortisol (OR 1.230, 95% CI [1.004–1.506]; *P* = 0.046), and androstenedione (OR 1.375, 95% CI [1.043–1.813]; *P* = 0.024) were associated with ACC (**Table 8**). We calculated the cut-off for the following four variables: tumor diameter larger than 36.00 mm, concentration of 11-deoxycortisol larger than 1.99 nmol/l, androstenedione higher than 3.01 nmol/l and BMI lower than 23.24 kg/m^2^.

**Table 8:** Univariate logistic analysis for the risk factors of ACC.

Variable	OR (95% CI)	*P*-value
BMI, kg/m^2^	0.738 (0.594–0.918)	0.006
Tumor diameter, mm	1.079 (1.038–1.121)	<0.001
11-deoxycortisol, nmol/l	1.230 (1.004–1.506)	0.046
Androstenedione, nmol/l	1.375 (1.043–1.813)	0.024
Progesterone, nmol/l	0.997 (0.948–1.049)	0.904

ACC, adrenocortical carcinoma; BMI, body mass index; OR, odds ratio; 95% CI, 95% confidence interval.

**Figure 3:**
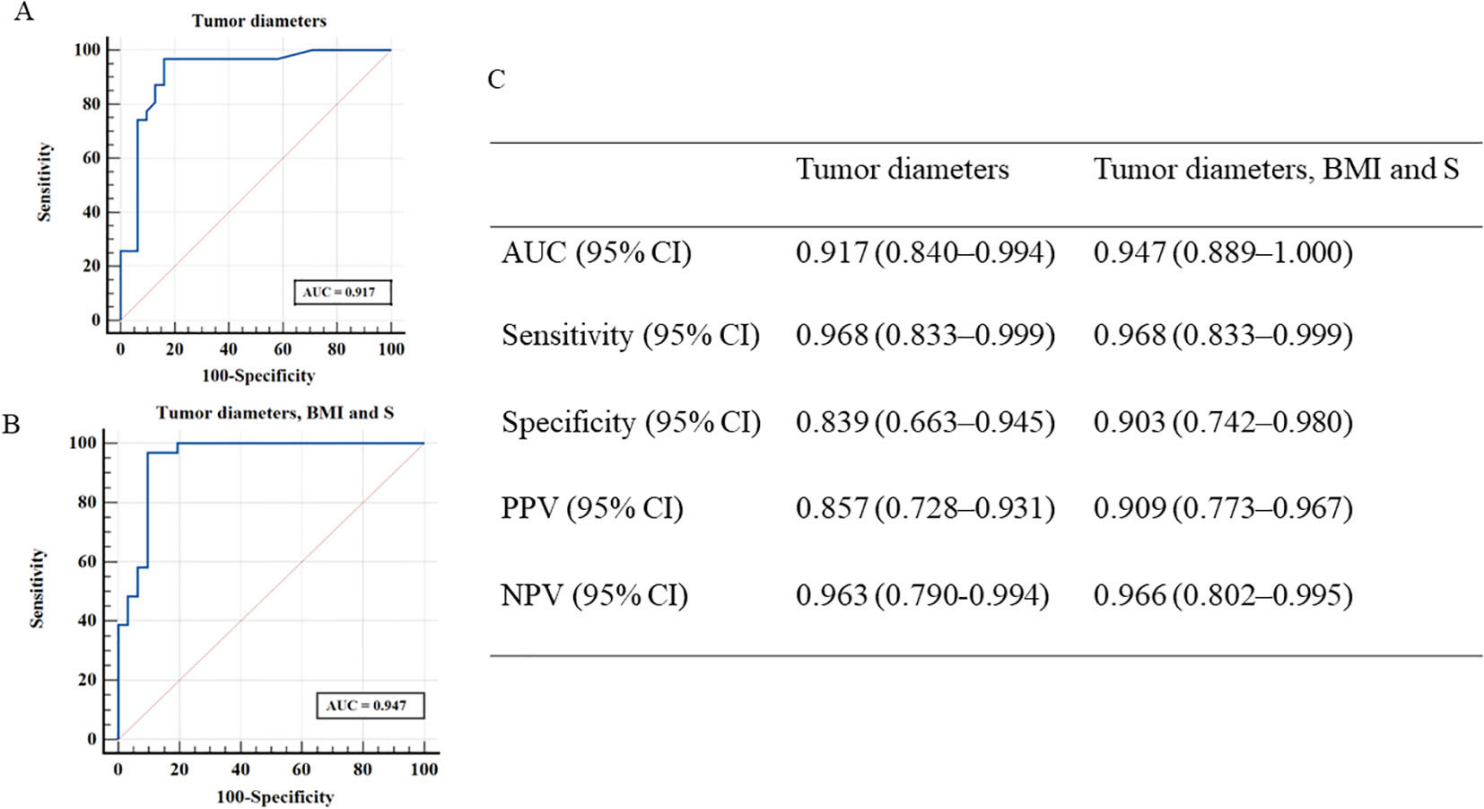
Receiver operating characteristic curves derived from discriminant analyses according to two different models [**(A)**, tumor diameters; **(B)**, tumor diameters, BMI and S]. **(C)** shows the measures of diagnostic performance derived from discriminant analyses for these two models for distinguishing patients with ACC and ACA. ACC, adrenocortical carcinoma; ACA, adrenocortical adenoma; S, 11-deoxycortisol; BMI, body mass index; NPV, negative predictive value; PPV, positive predictive value.

The AUC of tumor diameter was 0.917 (95% CI, 0.840–0.994) with a sensitivity of 96.8% (95% CI, 0.833–0.999), specificity of 83.9% (95% CI, 0.663–0.945), PPV of 85.7% (95% CI, 0.728–0.931) and NPV of 96.3% (95% CI, 0.790–0.994) (**Figures 3A, C**). The AUC of tumor diameter, 11-deoxycortisol, and BMI was (0.947, 95% CI [0.889–1.000]) (**Figures 3B, C**) better than that of a single index of tumor diameter, but failed to reach significance, with a sensitivity of 96.8% (95% CI, 0.833–0.999), specificity of 90.3% (95% CI, 0.742–0.980), PPV of 90.9% (95% CI, 0.773–0.967), and NPV of 96.6% (95% CI, 0.802–0.995). When considering BMI and 11-deoxycortisol separately for the diagnosis of ACC, the AUC for BMI alone was 0.729 (95% CI, 0.600–0.858), with a specificity of 77.4% (95% CI, 0.589–0.904) and sensitivity of 64.5% (95% CI, 0.454–0.808); however, with 11-deoxycortisol alone, the AUC was 0.689 (95% CI, 0.556–0.823), with a specificity of 64.5% (95% CI, 0.454–0.808) and sensitivity of 71.0% (95% CI, 0.520–0.858).

### Prognosis model of ACC

**Figure 4:**
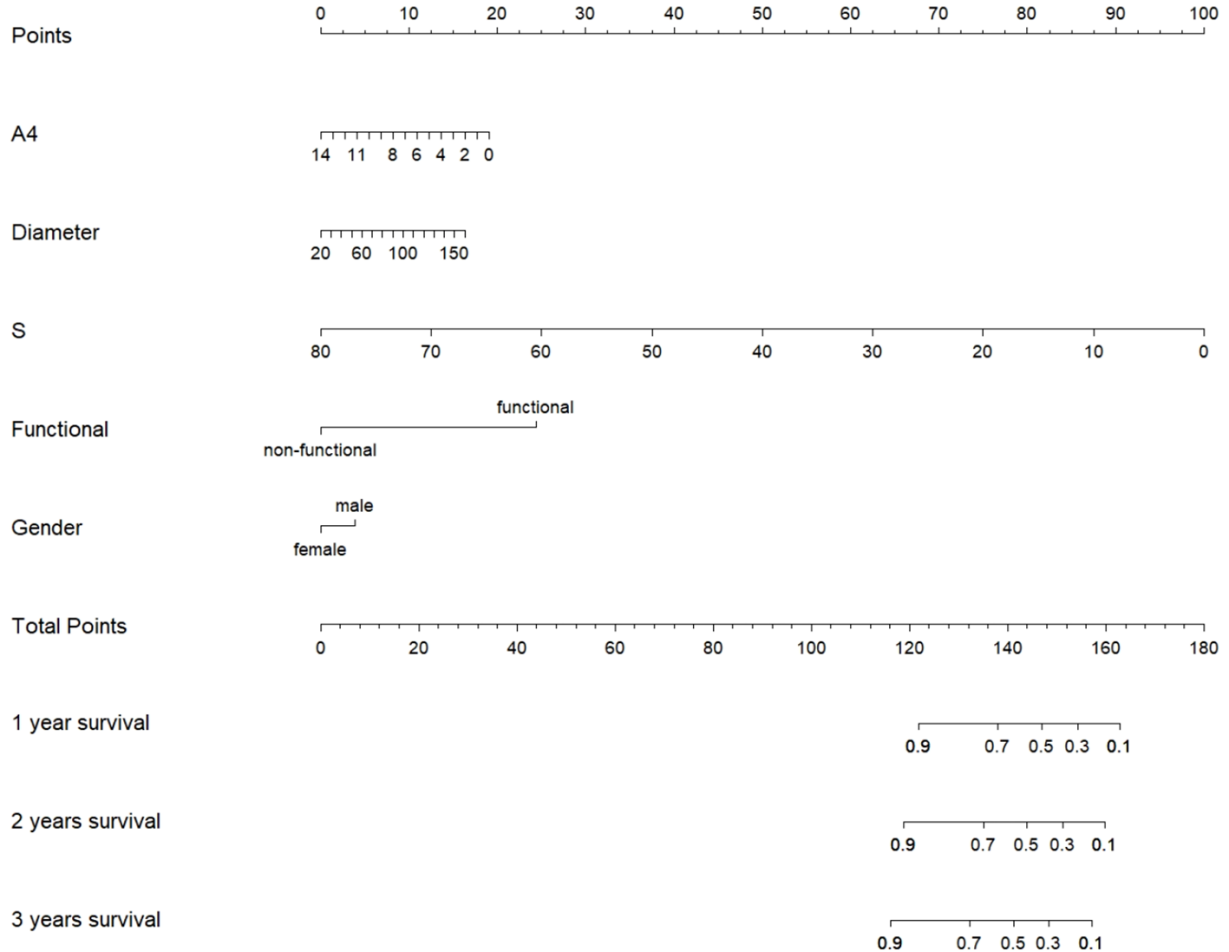
The nomogram was constructed based on five independent prognostic factors.

Considering that sex and functional status were observed to affect hormone profiles in this study, we included not only tumor diameter, 11-deoxycortisol, and androstenedione, but also sex and functional status in the prognostic model. We then used the five variables to build a nomogram for individualized prediction of patient of overall survival, where each level of every variable was assigned a point (**Figure 4**). By adding the points for all selected variables, the total number of points was obtained, and the probability of overall survival for a given participant was estimated. The median follow-up of the patients was 39.74 (29.11–50.38) months in this study. We found that male sex, functional tumors, larger tumor diameters, lower levels of 11-deoxycortisol, and lower levels of androstenedione were risk factors for lower overall survival.

## Discussion

ACC is a rare malignancy that arises from the adrenal cortex and has a poor prognosis due to its aggressive nature and unresponsiveness to conventional chemotherapeutic strategies. Therefore, it is imperative to identify biomarkers that can differentiate ACC from ACA at an early stage. In this study, we reaffirmed that serum 11-deoxycortisol was the most discriminative marker for all ACC cases. When combined with the tumor diameter, it proved to be highly effective in discriminating ACC with high sensitivity and specificity. However, when considering other steroid hormones and precursors, it is crucial to consider the functional status and sex. Elevated levels of progesterone, DHEA, and DHEAS in cortisol-secreting tumors strongly indicate ACC, particularly in functional tumors. Moreover, higher androstenedione levels in female adrenal tumors also suggest malignancy, especially in postmenopausal female.

In our study, 11-deoxycortisol levels were consistently confirmed to be higher and remained robust across a series of subgroup analyses, regardless of sex and functional status. Previous studies revealed a characteristic accumulation of 11-deoxycortisol rather than end products of adrenal steroidogenesis in ACC (10, 22–25). Taylor et al. recommended a 13-steroid panel with 11-deoxycortisol as the best marker, in which cohort only 10 ACC patients enrolled (11). Although a high heterogeneity of steroid secretion is commonly detected in these tumors, serum 11-deoxycortisol is the most sensitive indicator. Previous studies have reported a lack of expression of 11-β-hydroxylase (CYP11B1), which converts 11-deoxycortisol into active cortisol (26). The observed higher levels of 11-deoxycortisol indicated possible dysfunction of CYP11B1 (27, 28). Furthermore, only 11-deoxycortisol was elevated in the non-functional ACC compared to the non-functional ACA. Therefore, it is better to measure 11-deoxycortisol levels if it encounters a non-functional adrenal adenoma, which may help identify malignant adrenal tumors. In addition, serum 11-deoxycortisol was reported to be the most sensitive marker for predicting the recurrence and progression of ACC even earlier than imaging (29).

ACC is heterogeneous, and steroid production, especially sex hormones and their precursors, differs with sex, age, and functional status (30). We noticed that DHEA and DHEAS were increased only in the functional ACC group, which was usually low in benign cortisol-producing adenomas, due to suppression of ACTH (**Table 2**) (31–36). The levels of DHEAS and DHEA were higher in the functional ACC, indicating that they were not regulated by ACTH in the ACC. In addition, the expression of Phosphatidic Acid Phosphatase 1 enzyme (PAPSS1) is higher in functional ACC than in CPA, which may lead to the synthesis of DHEAS. In addition, an increase in organic anion transporter 4 (OAT4; also known as SLC22A11) was observed in the H295R cell line, facilitating the transportation of DHEAS into the blood (37). Previous studies have recognized that progesterone was increased in ACC patients (12, 13), but they did not consider functional status. We further demonstrated that high levels of progesterone were only observed in functional ACC compared to ACA subgroups. Consistent with our findings, Suzuki et al. observed that progesterone levels were elevated in cortisol-producing ACC (14). Thus, we speculate that progesterone, DHEA, and DHEAS are important index elements of diagnostic malignant factors only in functional ACC.

Previous studies have shown that androstenedione levels are significantly higher in ACC patients than in those with ACA (13). Our subgroup analysis further demonstrated that this difference was only observed in female ACC patients, especially in postmenopausal female, but not in male ACC patients. Consistent with our study, Schweitzer et al. showed that there was no significant difference in androstenedione levels between male ACC and ACA; the addition of androstenedione into the model did not significantly improve the predictive ability for male ACC. However, androstenedione has been shown to be an important factor in different predictive models of female ACC (12). Previous studies have suggested that androstenedione and other androgens decline in postmenopausal women; however, in premenopausal women androstenedione is secreted by the adrenal fascia (50%) and ovarian stroma (50%) (38). This suggests that postmenopausal women with elevated androstenedione levels and adrenal incidentalomas should be evaluated for the possibility of malignancy. We highlight that it is important to consider sex differences when analyzing androgen precursors in ACC patients.

In our study, clinical data were also informative. We found that a cut-off for adrenal tumors of 36 mm diameter, quite close to the clinical guideline recommendation of 40 mm (6), was the best in discriminating between ACC and ACA (sensitivity of 96.7% and specificity of 83.7%). Given that the patients were primarily staged ENSAT III–IV (64.4%) and the growth velocity of these tumors seemed to be very rapid, this cut-off may not be helpful in early diagnosis. Lower BMI, a classical malignancy-associated symptom, was suggestive of ACC in our study, regardless of functional status and sex. Advanced stage and coexisting malnutrition were the main contributing factors. The two clinical characteristics were non-specific when used alone, but when combined with other malignant characteristics, the specificity was improved compared to single factors. When we encounter a patient with a smaller adrenal mass together with other malignant characteristics on imaging, mixed one or two hormone hypersecretions, vigorous surgery may be advisable; if not, then more frequent follow-up is needed.

One strength of our study is its reliance on matching ACC patients with ACA patients based on sex, age (within ±5 years), and functional status to minimize tumor and patient heterogeneity in the serum steroid profiles. However, as this was a single-center retrospective study with a small sample size and predominantly stage III–IV patients, potential biases may have influenced the generalizability of the results and limited its value for early diagnosis. Second, the imaging data were incomplete, as some patients underwent CT scans while others underwent MRI. Further research incorporating multiple indices, including clinical data, imaging results, and steroid precursors, will be valuable for predicting ACC at an early stage. Third, we only detected hormone levels in fasting blood samples collected in the morning, without collecting multiple samples at different time points to observe changes in hormonal circadian rhythms. From this perspective, 24-hour urinary steroid metabolites might have an advantage due to their sensitivity to higher steroid secretion and disturbed circadian secretion after cancer. In fact, 24-hour urinary steroid metabolites have demonstrated their value in predicting ACC (22). However, the collection of 24-hour urine samples is cumbersome for patients and prone to sampling errors, with the proportion of incomplete collections being as high as 30% or more (39–41). Currently, it is difficult to determine whether serum steroid profiles or urine steroid metabolites are better for diagnosing ACC because no studies have directly compared serum or plasma steroids with urine steroid metabolites in ACC patients.

In conclusion, multi-steroid profiling by LC-MS/MS proves valuable in the preoperative discrimination between ACC and benign ACA, with the abundance of 11-deoxycortisol emerging as the most useful biomarker. Additionally, elevated levels of progesterone, DHEA, and DHEAS in cortisol-producing tumors, and increased androstenedione in female adrenal masses suggest malignancy. However, given the highly heterogeneous nature of ACC, these innovative approaches must undergo rigorous validation through large prospective clinical studies.

## Data Availability

The raw data supporting the conclusions of this article will be made available by the authors, without undue reservation.
